# Mid-term progressive loosening of hydroxyapatite-coated femoral stems paired with a metal-on-metal bearing

**DOI:** 10.1186/s13018-019-1271-1

**Published:** 2019-07-19

**Authors:** Trevor Gascoyne, Bryan Flynn, Thomas Turgeon, Colin Burnell

**Affiliations:** 1 Orthopaedic Innovation Centre, 320-1155 Concordia Avenue, Winnipeg, R2K2M9 Manitoba Canada; 2 Concordia Joint Replacement Group, 310-1155 Concordia Avenue, Winnipeg, Manitoba R2K 2M9 Canada; 3 Department of Surgery, University of Manitoba, Winnipeg, Manitoba Canada

**Keywords:** Hip arthroplasty, Aseptic loosening, Metal-on-metal, Hydroxyapatite, Radiolucency, Wear

## Abstract

**Background:**

Several hydroxyapatite (HA)-coated femoral stems from a single manufacturer were identified to have aseptically loosened at mid-term follow-up despite prior radiographic appearance of osseointegration. Possible causes and associated risk factors for stem loosening were explored through radiographic review and implant retrieval analysis.

**Methods:**

Forty-six retrieved hip stems (Corail, DePuy-Synthes) were identified and grouped by bearing type: metal-on-metal (MoM), metal-on-polyethylene, and ceramic-on-ceramic. Stem lucency was graded on post-operative radiographs up to the time of revision. Stems were examined for stripping of the HA coating, taper corrosion, and bearing wear in metal-on-metal cases. Patient demographics, implant design features, and perioperative data were collected from electronic databases and patient charts.

**Results:**

Aseptic loosening occurred in 37% of cases examined. MoM bearings were associated with 7.25 times greater risk of loosening compared to other bearing types. Stem radiolucency was more prevalent for MoM cases and, although not statistically significant, demonstrated progressive lucency. Taper corrosion appeared more severe for MoM cases and correlated with proximal stem radiolucency. Removal of the HA coating from the stems was associated with both taper corrosion and MoM bearing wear. Length of implantation was a confounding factor for the MoM cases.

**Conclusion:**

This study has demonstrated a high risk of mid-term loosening of previously osseointegrated HA-coated femoral stems when paired with a MoM bearing. The mechanism of loosening appears progressive in nature and related to the MoM bearing, possibly interacting with the HA coating. If such loosening is recognized early, rapid revision may allow for retention of the femoral stem.

## Background

Long-term survival of total hip arthroplasty (THA) requires excellent fixation between the host bone and the prosthesis. This fixation can be achieved via cement, bone in-growth, or bone on-growth. The latter can be promoted through the use of bone growth promoting substances, such as hydroxyapatite (HA). HA is a naturally occurring mineral found within bone composition, and its presence on the femoral component supports osseointegration of the stem [1, 2]. While primary stability of the femoral stem in cementless THA models depends on a press fit between the bone and femoral component, long-term stability requires osseointegration of cancellous bone to the HA-coated stem [3]. Hydroxyapatite-coated femoral stems demonstrate high clinical success rates [1, 4]; however, some concerns remain over the resorption of HA and delamination of HA from the stem which can lead to loosening of the implant [5].

In metal-on-metal (MoM) hip arthroplasty, bearing surfaces of the femoral head and acetabular liner are manufactured with high precision to allow fluid-film lubrication to minimize wear. Despite this design, both patient and implant-specific factors have been identified that can lead to significant bearing wear with production of large quantities of cobalt (Co) and chromium (Cr) wear particles and ions that can have local and systemic effects [6]. Metal wear particles induce an inflammatory response which can cause damage to surrounding soft tissue and bone [7, 8] resulting in pain, loss of function, and often revision arthroplasty [9]. Beyond local reactions, high systemic metal ion concentrations have been associated with poor osseointegration of the femoral stem. Shah et al. showed that high Co and Cr ions decrease osteoblastic activity and alkaline phosphatase activity, both important processes of bone growth and development [10]. Metal ions also have a direct negative effect on HA by binding into the HA crystal in place of calcium and inhibiting the mineralization process [11]. These deleterious effects of metal ions reportedly discourage initial osseointegration processes; however, the mid- to long-term effects on bone-HA fixation are not known and numerous confounding factors may also be of direct influence.

As a result of the poor clinical performance of MoM THAs, most manufacturers have eliminated MoM as a bearing option corresponding to steep decline in their use since 2008 [12, 13]. However, many tens of thousands of patients still have functioning MoM THAs and may yet be affected by the long-term effects of these generally poor performing implants.

Several HA-coated femoral stems were identified through the implant retrieval analysis program at the Orthopaedic Innovation Centre which had clinically loosened after several years of appearing radiographically osseointegrated. We hypothesized that these cases may be associated with metal particle generation from the MoM articulation or from corrosion of the head-neck taper and subsequent depletion of the HA coating on the femoral stem. Other possible factors of mid-term HA-coated femoral stem loosening, such as stress shielding, were not examined in this study.

This study was undertaken to examine the possible causes and associated risk factors for mid-term (3–7 years post-surgery) aseptic loosening of HA-coated femoral stems and to detail their clinical manifestation through radiographic and implant retrieval analysis.

## Methods

### Clinical data

This study is a retrospective analysis of patient charts, radiographs, and retrieved implants of a single femoral stem design, Corail® (DePuy-Synthes, Warsaw, IN). All HA-coated femoral stems were extracted from a regional implant retrieval database, resulting in a total of 46 retrievals. Research ethics approval was granted by the local review board (H2013:325, University of Manitoba). All retrieved implants were revised between 2007 and 2015 and consisted of 14 standard and 32 collared designs. Three study groups were created based on bearing type: metal-on-metal (MoM; *n* = 10), metal-on-polyethylene (MoP; *n* = 24), and ceramic-on-ceramic (CoC; *n* = 12). Patient data was collected for all retrievals, including age at primary surgery, length of implantation, reasons for revision, body mass index, femoral head offset, implant sizes, and head-neck taper size. The dataset generated from this study is available from the corresponding author on reasonable request.

### Radiographic review

**Fig. 1:**
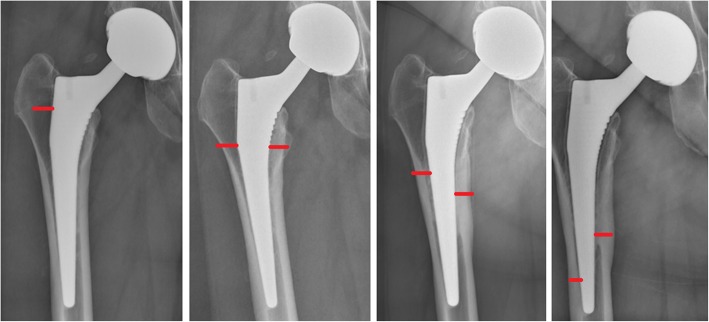
Example case of progressive radiolucency around a Corail stem with a metal-on-metal bearing over a period of 4 years. Red horizontal lines indicate the depth of radiolucency on the medial and lateral sides of the stem

All available post-operative radiographs were examined by a fellowship-trained arthroplasty surgeon (BF) for evidence of radiolucent lines around the femoral stem. The width and location of each lucent line were documented according to femoral Gruen zones for all available radiographs of each patient [14]. The incidence, location, and width in millimeters of radiolucent lines were summed for each follow-up radiograph and each case examined for evidence of progression (Fig. 1).

### Retrieval analysis

**Fig. 2:**
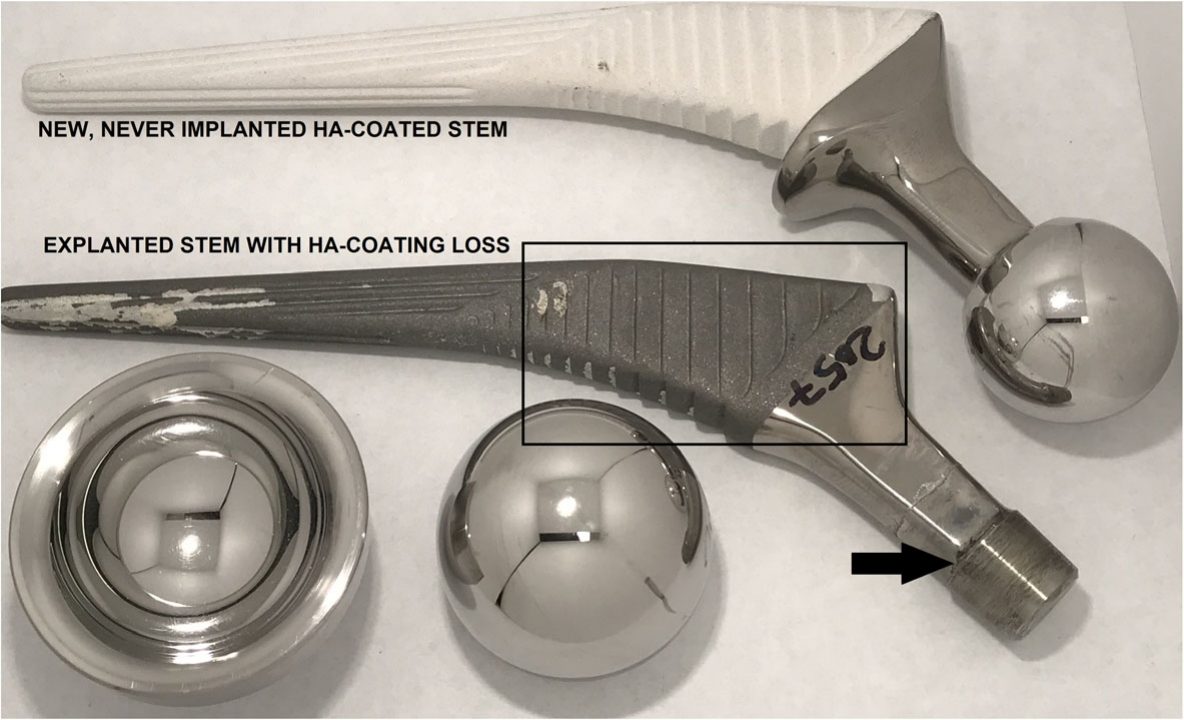
Example case of HA coating removal on an explanted Corail stem with metal-on-metal bearing adjacent to an “as manufactured” Corail stem with intact HA coating. Arrow indicates the area of taper corrosion. Rectangle indicates the proximal region of the stem

Retrievals were analyzed in detail for evidence of taper damage, HA coating loss (Fig. 2), and bearing wear. Femoral head-neck taper surfaces were qualitatively graded for corrosion following the Goldberg method [15]. Removal of HA from the femoral stem was graded 0–3 based on the observed area of HA coating loss: 0 = no HA removal, 1 = 1–33% HA removal, 2 = 34–66% HA removal, and 3 = 67–100% HA removal. The location of HA removal was defined proximally and distally as it appeared from radiographic analysis that stem loosening progressed in a top-down fashion.

Bearing wear and taper corrosion damage was measured only on MoM retrievals, as insignificant amounts of wear or corrosion were noted in the CoC and MoP groups and the quantification of metal debris released in vivo was of prime interest to this study. Measurement of the taper surfaces (head and neck independently) was performed with a coordinate measurement machine (CMM, Zeiss Prismo, etc) following the ASTM F3129 [16] standard. Virtual models of each tapered surface were created based on high-density point scans (< 0.1 mm point spacing), and material removal (corrosion) was computed using a Boolean subtraction with the ideal “as manufactured” geometry of the taper surface.

In a similar fashion, bearing wear was measured on both the head and liner of all MoM retrievals, except with a maximum point spacing of 0.25 mm, due to the larger surface area of the bearings. Virtual models were created as were perfect spheres to represent the “as manufactured” surface, based on the method described by Hoffmann and Pfirrmann [17]. In short, this method involves the truncation of points within the worn regions of the bearing based on a histogram of radial distances of every measured point. By removing points in the worn regions, an idealized sphere can be created that is best fit to the remaining surface of the bearing, assumed to be largely unworn. Volumetric bearing wear was computed using a Boolean subtraction between the measured surface and the ideal sphere.

### Statistical analysis

Statistical analysis was performed using SAS (Statistical Analysis Software v9.3, Cary, NC). Analysis of variance was applied to test differences between bearing groups. Pearson’s correlations were established between patient and implant variables. Fisher’s exact test was used to calculate odds ratios and relative risk of MoM THR for aseptic loosening. Linear regression was applied to radiographic data to determine the rate of progression of radiolucency. Statistical significance was set at *p* < 0.05.

## Results

### Clinical data

Aseptic loosening occurred in 17 of 46 cases that were examined (Table 1). Metal-on-metal bearings were associated with increased risk of aseptic loosening (odds ratio 7.25, 95% CI 1.54–34.10, *p* = 0.020). Femoral head size, head offset, collared femoral stem, and patient age were not associated with aseptic loosening (*p* > 0.05). Mean time to revision for MoM was 4.9 years (range 3.0–7.0) compared to 1.6 years (range 0.1–9.3) for other bearing couples (*p* = 0.004). Body mass index was not statistically different between bearing groups nor between patients having a MoM articulation and revised for aseptic loosening versus the remainder of the cohort (*p* = 0.844).

**Table 1:** Patient demographics and reasons for revision separated by bearing group

Bearing	*N*	Collared stems	Age (SD)	Implantation time (SD) years	Gender	BMI (SD) kg/m^2^	Reasons for revision
MoM	10	0	52 (12)	4.9 (1.3)	5 M: 5F	30.9 (4.0)	Aseptic loosening (7) Periprosthetic fracture (2) High metal ions (1)
CoC	12	5	58.9 (6.4)	1.8 (1.7)	6 M: 6F	36.3 (13.2)	Infection (5) Aseptic loosening (4) Periprosthetic fracture (2) Instability/malposition (1)
MoP	24	9	71.6 (11.6)	1.2 (1.6)	11 M: 13F	31.7 (7.0)	Periprosthetic fracture (9) Infection (8) Aseptic loosening (6) Dislocation (1)

### Radiographic evaluation

Radiolucency surrounding the proximal stem region was more prevalent in the MoM group compared to CoC and MoP (Table 2). However, this difference did not reach statistical significance. Presence and thickness of proximal radiolucent lines in all available radiographs were assessed for 9 of 10 MoM, 10 of 12 CoC, and 17 of 24 MoP patients with their latest radiographic follow-up occurring at 49.0, 18.8, and 20.5 months after index surgery, respectively. Collared stems were not associated with radiolucency of the proximal stem (*p* = 0.637). Proximal radiolucency was found to advance at a mean rate of 0.9 mm/year in the MoM group.

**Table 2:** Retrieval analysis and radiographic evaluation for each bearing group

Bearing group	Goldberg corrosion score (0–3 scale)	HA coating removal (0–3 scale)		Mean thickness of radiolucent lines at latest follow-up	
		Proximal	Distal	Proximal (Gruen zones 1 and 7)	Distal (Gruen zones 3, 4, and 5)
MoM	2.1 (0.8)^*^	2.6 (0.5)	2.1 (0.9)	2.9 (1.7)	0.4 (1.3)
CoC	0.8 (0.9)	1.4 (0.8)	1.7 (0.9)	1.7 (1.6)	0
MoP	0.4 (0.9)	1.0 (1.0)	0.8 (0.9)	1.5 (1.7)	0.6 (1.5)

^*^Statistical significance (*p* = 0.001)

### Retrieval analysis

The presence and observed severity of taper corrosion was significantly higher in the MoM group compared to all others (Table 2). Proximal and distal HA coating removal was similarly greater in the MoM group; however, this was not statistically significant. Stem design (collared or non-collared) was not associated with HA coating removal (*p* = 0.267).

Removal of proximal HA coating was strongly associated with taper corrosion score and mildly associated with the total measured metal that was released from the THA (Table 3). Proximal radiolucency also showed weak correlation with taper corrosion score, yet did not display a statistically significant association to proximal HA coating loss. Implantation period was found to correlate significantly with HA coating removal and radiolucency (both proximal and distal) as well as taper corrosion score.

**Table 3:** Statistically significant correlations between clinical, radiographic, and retrieval variables for all bearing groups

Variable	Variable 2	*R*	*p* value
Implantation period	HA coating removal (proximal)	0.643	< 0.001
	HA coating removal (distal)	0.597	< 0.001
	Goldberg score	0.529	< 0.001
	Radiolucency (proximal)	0.359	0.034
	Radiolucency (distal)	0.351	0.039
HA removal (proximal)	HA Removal (distal)	0.539	< 0.001
	Goldberg score	0.586	< 0.001
Goldberg score	Radiolucent lines (proximal)	0.357	0.048

Measured bearing wear in the MoM group averaged 3.3 mm^3^ cumulative for heads and liners, with the majority of wear occurring on the femoral head (Table 4). Similarly, taper corrosion damage (volume) occurred primarily on the femoral head taper with a mean of 1.80 mm^3^ cumulative material removal (Table 4). Total metal release was correlated to proximal HA coating removal in the MoM group (*R* = 0.794, *p* = 0.011).

**Table 4:** Bearing wear and taper corrosion volumes measured on retrieved MoM bearings

MoM patient	Head wear (mm^3^)	Liner wear (mm^3^)	Taper corrosion score	Head corrosion volume (mm^3^)	Neck corrosion volume (mm^3^)	Total metal release (mm^3^)
Pt 1	1.76	–	–	0.46	0.20	2.42
Pt 2	1.14	–	2	0.15	0.33	1.62
Pt 3	0.78	0.40	1	0.71	0.50	2.39
Pt 4	8.77	0.50	2	1.13	0.31	10.71
Pt 5	0.84	0.15	3	7.10	0.25	8.34
Pt 6	1.63	0.62	3	1.23	0.15	3.63
Pt 7	1.35	0.07	1	0.21	0.17	1.80
Pt 8	2.01	0.82	–	0.87	0.30	3.99
Pt 9	9.85	0.32	2	0.71	0.72	11.60
Pt 10	1.16	0.18	3	5.40	0.2	6.93
Mean (SD)	2.93 (3.40)	0.38 (0.26)	2.1 (0.8)	1.80 (2.41)	0.31 (0.18)	5.34 (3.77)

## Discussion

Despite improvements in bearing technology, aseptic loosening remains a leading cause for revision hip arthroplasty [18]. Promising wear simulator data for MoM has not translated clinically into improved survivorship, and results of the DePuy Pinnacle MoM THA have been varied. Some authors have noted good mid-term survivorship from 97.0–99.4% at 5–7 years [19–22]. Others have found much worse survival ranging from 92.8% at 7 years [23], 88.9% at 8 years [24], to 83.6–86% at 9 years [25, 26]. Ten-year cumulative percentage probability of revision reported in the Australian and UK national joint registries for the Corail stem and Pinnacle cup is 3.2–4.9% for conventional bearings vs 11.7–14.6% for MoM. Our results for Corail Pinnacle MoM suggest taper corrosion is a factor in the loosening of a fully HA-coated stem that has previously shown excellent survivorship with other bearings such as MoP, CoP, and CoC [27, 28].

A recent publication by Buttaro et al. described a similar phenomenon with this same stem design presenting with “metaphyseal debonding” at a mean of 36 months from surgery. Their case-control study found association with increased BMI and the use of hard-on-hard bearing surfaces (both ceramic-on-ceramic and MoM) [29]. In contrast, our study did not find patient variables, including BMI, to be associated with loss of HA coating, nor were ceramic-on-ceramic bearings found to undergo HA coating loss. Buttaro et al. were only able to assess 6 implants of the 18 cases identified, all six of which were either ceramic-on-ceramic or ceramic-on-polyethylene. Four of the 6 retrieved stems demonstrated loss of the proximal HA coating, but no analysis of the condition of the taper was discussed in the publication.

We found the presence and observed severity of taper corrosion to be associated with aseptic loosening of HA-coated femoral stems in a relatively small number of samples. The predominant mechanism of taper corrosion is thought to be mechanically assisted crevice corrosion [15]. Other contributing factors include use of dissimilar alloys [30], taper size and design [31], head size and length [32], stem offset [33], flexural rigidity of the stem neck [34], intraoperative contamination with blood or fluid, assembly force [35], and length of implantation [33, 36]. The Corail taper with its short, roughened surface may be particularly prone to corrosion [37]. Interestingly, MoP and CoC had no difference in corrosion scores. Dissimilar metals alone fail to account for the difference seen among the MoM as the taper, and CoCr head of the MoP group had little corrosion, although this may be explained by their shorter implantation time [32].

Corrosion score was also positively correlated with proximal HA loss. All retrieved MoM stems in our study had significant proximal HA loss (grade 2 or 3), with six out of seven stems having failed for aseptic loosening also showing a similar degree of HA loss. The proposed mechanisms of HA loss in the literature include delamination from the metal substrate, dissolution into extracellular fluid, and cell-mediated resorption. Based on an autopsy retrieval study of HA-coated stems, Bauer et al. suggested osteoclastic resorption was the primary mode of HA resorption, assisted by enzymatic breakdown common to bone remodeling [38]. This process may be enhanced by corrosion byproducts that act as potent activators of macrophage-induced osteolysis [39]. Additionally, delayed-type hypersensitivity reactions have been implicated in aseptic osteolysis seen in MoM due to metal-induced lymphocytes releasing cytokines to shift the balance in favor of osteoclastic resorption [40]. Three of the MoM cases that failed for aseptic loosening in our series had tissue pathology results available. The tissue analyses were consistent with mild to moderate hypersensitivity reaction with moderate ALVAL scores (6, 7, and 7), suggesting this may have contributed to stem loosening.

Another possible explanation relating proximal HA loss to taper corrosion is that HA can be dissolved in acidic solutions, as demonstrated in dentistry literature [41]. While the pH of the joint space is more neutral compared to that of the mouth, it is feasible that it is locally altered by corrosion and wear products. Cobalt chromium and titanium alloys are biocompatible metals primarily because when in contact with oxygen, they form a protective surface film of oxidized metal rendering it non-reactive. Mechanical removal of this layer either by taper micromotion or bearing wear results in immediate reformation of the oxide layer with nearby oxygen atoms from water molecules. This oxidation reaction creates free H^+^ ions which increase the acidity of the surrounding environment. Milosev et al. measured the pH of osteoarthritic synovial fluid, compared it to those with metal hip and knee arthroplasty components, and noted a small but significant decrease in pH in the arthroplasty group [42]. Inflammatory conditions such as rheumatoid arthritis and septic arthritis have also been found to lower synovial fluid pH. Willert et al. measured the pH at the interface of 17 titanium alloy stems at time of revision noted to have signs of corrosion and found 7 stems had sub-physiologic levels dropping as low as 2.2 [43]. Lowering of local pH by continual reoxidation of CoCr alloy on various surfaces of the THA, and specifically the taper, can therefore potentially contribute to dissolution of the HA coating and aseptic loosening.

A further explanation for stem loosening is increased particulate debris from bearing wear, which has been found in some MoM implants that have higher risk of mid-term aseptic loosening. When functioning well, MoM bearings are extremely wear resistant; however, they are less tolerant for implant malposition and the smaller size and subsequent greater surface area of wear debris has high bioactivity and cytotoxicity. Further, smaller cup size has been shown to have a higher risk of wear in MoM bearings as the corresponding metal insert is thinner with higher likelihood of deformation when engaged in the shell taper. The 50 mm and 52 mm outside diameters of 36 mm liners (wall thickness of 7 mm and 8 mm) have demonstrated significantly increased rates of revision in the literature [25]. In our series, liner wall thickness ranged 7 mm up to 12 mm, with one sample at 7 mm and two samples at 8 mm. Insufficient bearing clearance has also been implicated as a possible cause for premature failure of MoM THAs [25]. Reduced clearance increases likelihood and severity of bearing wear on MoM THAs but may also increase the toggling torque on the head-neck taper. Micromotion of the head-neck taper initiates mechanically-assisted crevice corrosion, a well-described process of taper corrosion and subsequent metal debris release [15]. While increased torque-induced stem micromotion may lead to failure of initial press fit and osseointegration as suggested in Pilliar et al., [44], it does not seem to explain the progressive loosening of previously well-fixed stems seen in the current series.

Higher cup inclination angle in MoM bearings [45] has been shown to influence wear; however, this was unlikely a factor in our study as mean cup inclination of the 7 MoM hips that failed for aseptic loosening with available x-rays was 42°(range 39–47°). Reinisch et al. found no evidence of increased bearing wear among 22 single manufacturer MoM implants with 28 mm heads revised for early (12–59 months) aseptic loosening, suggesting this process can occur under normal bearing wear settings [46]. Langton et al. also found 6 cases of failed MoM with well-functioning bearing surfaces and described significantly worn taper junctions as the cause [47]. This finding has been confirmed in a retrospective cohort study examining mid-term revisions of the pinnacle MoM bearing paired with Corail or S-ROM stems showing failure from the taper junction was more common than at the bearing surface [25]. The MoM bearing wear in our study was similarly found to be low and within the normal range of reported values for this articulation [25].

Among the nine MoM hips that had 2 or more x-rays available for review, radiolucency progressed from proximal to distal at a mean rate of 0.9 mm/year. None of the cases saw progression stop until time of revision. If radiolucency progression is related to ongoing taper corrosion that predictably leads to mid-term aseptic loosening, then this represents both an early warning sign and a potential therapeutic window. If recognized early when the stem remains well fixed, removing the source of corrosion may salvage the stem, limit soft tissue damage, and avoid the morbidity of more extensive revision. A recent systematic review identified nine studies examining the treatment of hip implant failures secondary to taper corrosion with the majority being treated with stem retention and exchange to a ceramic head with titanium sleeve [48]. Although follow-up was short (1–36 months), the majority of patients had resolution of their symptoms.

Limitations of this study include the small sample size available, x-ray availability in only a subset, varying demographics between the patient groups (age, demand on the implant, and potentially activity level), variation in implant dimensions and designs, and longer implantation time in the MoM group which biases radiolucency, HA coating removal, and taper corrosion. The observed mid-term loosening, however, is unexpected in a stem with good, lengthy clinical track record.

## Conclusion

Our retrieval study of a fully HA-coated stem from a single manufacturer showed a higher risk of mid-term aseptic loosening when paired with a MoM bearing. Loosening was correlated with the presence and severity of taper corrosion which was significantly higher with MoM compared to other bearing groups. Taper corrosion scores were also correlated with HA removal from the stem. This may be related to a cell-mediated response, lowering of local pH, or both. Radiolucency progressed at an average rate of 0.9 mm/year until the time of revision. Corrosion should be considered as a cause of aseptic loosening of fully HA-coated stems, and if recognized early, revision is recommended.

## Data Availability

The dataset generated from this study is available from the corresponding author on reasonable request.

## Abbreviations

BMI: Body mass index, the ratio of a person’s body weight in kilograms versus their height in meters (weight/height^2^)
CoC: Ceramic-on-ceramic, refers to the bearing materials of the ball and socket joint of the artificial hip
HA: Hydroxyapatite, refers to a calcium phosphate-based coating applied to the femoral hip stem of interest in this study
MoM: Metal-on-metal, refers to the bearing materials of the ball and socket joint of the artificial hip
MoP: Metal-on-polyethylene, refers to the bearing materials of the ball and socket joint of the artificial hip
SD: Standard deviation, the average difference between individual values and the group mean
THA: Total hip arthroplasty, refers to the surgery of replacing a person’s hip joint with artificial components
